# RNA sequencing analysis revealed the induction of CCL3 expression in human intracranial aneurysms

**DOI:** 10.1038/s41598-019-46886-2

**Published:** 2019-07-17

**Authors:** Tomohiro Aoki, Hirokazu Koseki, Haruka Miyata, Masayoshi Itoh, Hideya Kawaji, Katsumi Takizawa, Akitsugu Kawashima, Hiroshi Ujiie, Takashi Higa, Kenzo Minamimura, Toshikazu Kimura, Hidetoshi Kasuya, Kazuhiko Nozaki, Akio Morita, Hirotoshi Sano, Shuh Narumiya

**Affiliations:** 1 Innovation Center for Immunoregulation Technologies and Drugs (AK project), Kyoto University Graduate School of Medicine, Kyoto, 606-8501 Japan; 2 Department of Molecular Pharmacology, National Cerebral and Cardiovascular Center, Osaka, 565-8565 Japan; 3 Core Research for Evolutional Science and Technology (CREST) from Japan Agency for Medical Research and Development (AMED), Kyoto University Graduate School of Medicine, Kyoto, 606-8501 Japan; 4 Department of Neurosurgery, Tokyo Women’s Medical University Medical Center East, Tokyo, 116-8567 Japan; 5 Department of Neurosurgery, Shiga University of Medical Science, Shiga, 520-2192 Japan; 6 RIKEN Preventive Medicine and Innovation Program, Saitama, 351-0198 Japan; 7 Department of Neurosurgery, Japanese Red Cross Asahikawa Hospital, Hokkaido, 070-8530 Japan; 8 Department of Neurosurgery, Tokyo Women’s Medical University Yachiyo Medical Center, Chiba, 276-8524 Japan; 9 Department of Neurosurgery, Tokyo Rosai Hospital, Tokyo, 143-0013 Japan; 10 Department of Neurosurgery, Shinkawahashi Hospital, Kanagawa, 210-0013 Japan; 11 Department of Neurosurgery, NTT Medical Center Tokyo, Tokyo, 141-8625 Japan; 12 Department of Neurosurgery, Japanese Red Cross Medical Center, Tokyo, 150-8935 Japan; 13 Department of Neurological Surgery, Nippon Medical School, Tokyo, 113-8603 Japan

**Keywords:** Aneurysm, Translational research

## Abstract

Intracranial aneurysm (IA) is a socially important disease as a major cause of subarachnoid hemorrhage. Recent experimental studies mainly using animal models have revealed a crucial role of macrophage-mediated chronic inflammatory responses in its pathogenesis. However, as findings from comprehensive analysis of unruptured human IAs are limited, factors regulating progression and rupture of IAs in humans remain unclear. Using surgically dissected human unruptured IA lesions and control arterial walls, gene expression profiles were obtained by RNA sequence analysis. RNA sequencing analysis was done with read count about 60~100 million which yielded 6~10 billion bases per sample. 79 over-expressed and 329 under-expressed genes in IA lesions were identified. Through Gene Ontology analysis, ‘chemokine activity’, ‘defense response’ and ‘extracellular region’ were picked up as over-represented terms which included CCL3 and CCL4 in common. Among these genes, quantitative RT-PCR analysis using another set of samples reproduced the above result. Finally, increase of CCL3 protein compared with that in control arterial walls was clarified in IA lesions. Findings of the present study again highlight importance of macrophage recruitment via CCL3 in the pathogenesis of IA progression.

## Introduction

Intracranial aneurysm (IA) is a reginal bulging of intracranial artery usually at its bifurcation site and characterized with degenerative changes in walls and presence of inflammatory infiltrates. IA is a socially important disease because of not only the high prevalence in general public but also its potential to evoke subarachnoid hemorrhage after rupture. Here, as subarachnoid hemorrhage is the most severe form of stroke with the mortality rate of up to 50%^1^, treatment of IAs before rupture is essential for social health. Nevertheless, more than half of patients with IAs remain untreated^2^ because of the lack of medical treatment for this disease. Given the high prevalence of IAs and poor outcome once after rupture, development of medical therapy for IAs is socially demanded.

Recent experimental studies mainly using animal model of IAs have accumulated evidence about mechanisms underlying IA formation, progression and rupture^3–5^. Today, IA is defined as a chronic inflammatory disease of intracranial arteries presumably triggered by hemodynamic stress loaded and mediated/exacerbated by infiltrated macrophages^5–7^. However, although many have revealed about the contribution of chronic inflammation to the pathogenesis through extensive researches, comprehensive view of molecular machinery working to induce IA remains to be elucidated. Thereby, we have not yet convinced whether the focus to inflammatory responses as a major cascade regulating the pathogenesis has an adequate rationale. For this purpose, comprehensive gene expression profiles by microarray analysis, which is the widely-used and powerful tool, have also been obtained about IAs and reported^8–13^. Many of these studies mainly analyzed ruptured IAs dissected from patients with aneurysmal subarachnoid hemorrhage and demonstrated the up-representation of inflammation-related cascade in common, supporting the involvement of inflammatory response in the pathogenesis of IA progression and especially rupture. However, because the increased intra-cranial pressure or excessive activation of synthetic nerve system after onset of subarachnoid hemorrhage greatly influences gene expression profile, careful interpretation should be paid. To more properly evaluate up- or down-representative cascades in IA lesions which may be involved in the pathogenesis of this disease and can be therapeutic target and to check whether inflammation-related cascades are indeed picked up, we have selectively collected human un-ruptured IA specimen and examined gene expression profile over-expressed in IA lesions compared with control arterial walls.

## Results

### Characteristics of human specimen harvested

We collected 55 IA specimen and control arteries from same patients (total 110 specimens) for RNA sequencing or quantitative RT-PCR analyses in the present study during neck clipping of unruptured lesions with written informed consent. Among 55 sample sets collected, 33 sample sets were actually used in the present study (3 for preliminary experiment to purify total RNA, 12 for RNA sequencing analysis, 18 for quantitative RT-PCR analysis). Notably, the average age and also the size of the present cases are closely similar with those reported in a Japanese large cohort (UCAS Japan)^2^ (average size; 5.8 mm (all cases), 5.2 mm (only cases subjecting to RNA sequencing analysis), 5.8 mm (only cases subjecting to quantitative RT-PCR analysis) in the present study, 5.7 mm in UCAS Japan, average age; 62.0 year-old (all cases), 62.1 year-old (only cases subjecting to RNA sequencing analysis), 63.1 year-old (only cases subjecting to quantitative RT-PCR analysis) in the present study, 62.5 year-old in UCAS Japan), suggesting that results obtained from the present study represent common pathophysiology of unruptured IAs not the specific population of them.

### Gene expression profile in IA lesions by RNA sequencing

Because human IA specimens were harvested during microsurgery, sometimes it took a while before stabilization of these dissected specimens resulting in the degradation of RNA. In addition, as arterial wall is stiff in nature, it is usually technical challenge to purify the adequate quality and quantity of RNA for analyses. Indeed, we purified total RNA from 12 IAs but only 4 of them met the criteria suitable for RNA sequencing analysis. The major reason to exclude 8 total RNA samples from an analysis is an amount of total RNA purified (less than 10 ng) due to degradation. Using these 4 RNA samples from IA lesions and 3 samples from control arteries of same patients (1 samples from the control artery was excluded because of the low yield.), we performed RNA sequencing analyses to obtain gene expression profile in an IA lesion compared with that in a control arterial wall. RIN (RNA Integrity Number) values of these sequenced samples were between 6.6 and 8.1 (Median; 7.5). Read count obtained in the present experiment was about 60~100 million per sample which yielded 6~10 billion bases (read length; 100 base pair). Among these reads, around 25 million fragments per sample were successfully mapped to the reference genome with only the minor contamination of ribosomal RNA (about 0.4~0.9 million) and its value was enough for further analyses.

**Figure 1:**
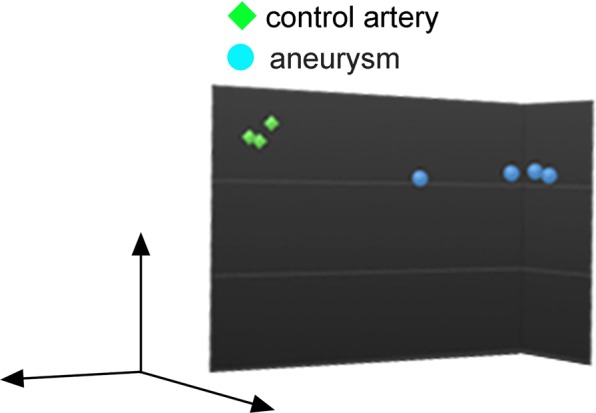
Principal component analysis of gene expressions in control arterial walls and intracranial aneurysms. Image from Principal component analysis of gene expressions in control arterial walls (n = 3) and intracranial aneurysms (n = 4) is shown, where genes expressed in primary culture of smooth muscle cells from human carotid artery were excluded.

In the preliminary analysis, majority of changes in gene expression profile in IA lesions compared with that in control arterial walls reflected loss of smooth muscle cells in lesions, which is the well-known histopathological character of IAs, making further analyses difficult. Indeed, about the five times more GO-terms were picked up as down-represented ones in IA lesions compared with that up-represented (Supplementary Tables S1 and S2) and the terms indicating the loss of smooth muscle cells occupied the top (Supplementary Table S1). Therefore, gene expression profile of commercially available primary culture of smooth muscle cells from human carotid artery (Supplementary Fig. S1) was first obtained and then genes with over 10 TPM (transcripts per million) in these cells were subtracted from the gene list of human IA lesions and control arterial walls obtained from RNA sequencing analysis. Because a primary culture of smooth muscle cells from human intracranial arteries was not available, we alternatively selected a primary culture derived from carotid artery. As the primary culture of smooth muscle cells rapidly proliferated when RNA was purified, this primary culture seems to be in the synthetic state. As a result of these procedures, we identified a couple of differentially expressing genes between IA lesions and control arterial walls. Principal component analysis demonstrated the cluster formation both in gene expression of IA lesions and control arterial walls and cluster of each group occupied completely different location in coordinate axis (Fig. 1), suggesting the presence of notably different gene expression pattern between groups; IA lesions and control arterial walls. We identified 79 over-expressed and 329 under-expressed genes in IA lesions compared with expressions in control arterial walls (Supplementary Tables S3 and S4). Here, under-expressed genes mainly involved smooth muscle cell-related ones, reflecting loss of these cells in IA lesions, and therefore, by gene ontology analysis (GO analysis), GO-terms related with loss of smooth muscle cells such as ‘muscle contraction’ in BP (biological process), ‘contractile fiber’ in CC (cellular component) and ‘structural constituent of muscle’ in MF (molecular function) were indeed picked up as down-representative ones (Supplementary Tables S5–S7). Notably, only 79 genes were identified as over-expressed ones in IA lesions (Supplementary Table S3), suggesting the nature of IA as a relatively silent (not active) lesion. Intriguingly, in all three categories, BP (Supplementary Table S8), CC (Supplementary Table S9) and MF (Supplementary Table S10) in GO-term, *CCL3*, *CCL4* and *CXCL10* were picked up as over-expressed ones (Table 1 and Supplementary Tables S11 and S12) and all of these genes coded chemoattractants for macrophages.

**Table 1:** Genes contained in up-represented terms in Gene Ontology analysis (Biological Process).

Symbol	Gene
*CLEC5A*	C-type lectin domain family 5, member A
*FCGR1A*	Fc fragment of IgG, high affinity Ic, Ia, receptor (CD64)
*CCL3*	chemokine (C-C motif) ligand 3
*CCL3L3*, *CCL3L1*	chemokine (C-C motif) ligand 3-like 3; chemokine (C-C motif) ligand 3-like 1
*CCL4*	chemokine (C-C motif) ligand 4
*CCL4L1*	chemokine (C-C motif) ligand 4-like 1; chemokine (C-C motif) ligand 4-like 2
*CXCL10*	chemokine (C-X-C motif) ligand 10
*C4A*	complement component 4A (Rodgers blood group)
*C4B*	complement component 4B (Chido blood group)
*DCDC2*	doublecortin domain containing 2
*HAMP*	hepcidin antimicrobial peptide

### Expression of selected genes in another set of human IA specimen by quantitative RT-PCR analysis

**Figure 2:**
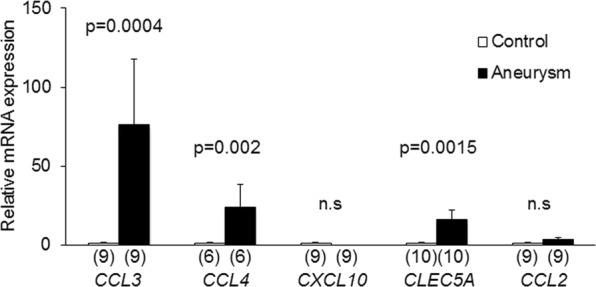
Induction of *CCL3*, *CCL4*, *CLEC5A* mRNA expression in intracranial aneurysm lesions. Total RNA was purified from dissected control arterial walls and intracranial aneurysm lesions and mRNA expressions of *CCL3*, *CCL4*, *CXCL10*, *CLEC5A* and *CCL2* were examined by quantitative RT-PCR analysis. The numbers of specimens analyzed are shown in parentheses. Statistical analysis was performed using a Mann-Whitney test. All bars indicate mean ± SEM. n.s; statistically not significant.

Next, to confirm the reproducibility of results from RNA sequencing analysis, quantitative RT-PCR analysis using RNA samples purified from another set of cases (18 cases) was done. In quantitative RT-PCR analysis, we selected *CCL3*, *CCL4*, *CXCL10* and *CLEC5A*, a C-type lectin expressed on macrophages, from over-expressed genes in RNA sequencing analysis as targets because the crucial role of macrophage-mediated inflammatory response in the pathogenesis of IA formation has been implicated^6,14,15^ in animal studies and consistently the presence of infiltrated macrophages in human lesions are demonstrated^16–18^. We found that expressions of *CCL3*, *CCL4* and *CLEC5A* were significantly over-expressed in IAs compared with those in control arterial walls from same patients consistently with the result in RNA sequencing analysis (CCL3; n = 9, p = 0.0004, CCL4; n = 6, p = 0.002, CLEC5A; n = 9, p = 0.0015) (Fig. 2). As to *CXCL10* expression, quantitative RT-PCR analysis failed to reproduce the result in RNA sequencing analysis; the significant increase in IAs compared with control arterial walls. Intriguingly, although contribution of MCP-1-mediated recruitment of macrophages in IA walls to the initiation of experimental IAs in mice has been demonstrated^14,15^, MCP-1 (*CCL2*) expression tended to be increased in IA walls but differences did not reach statistically significant (n = 10, p = 0.0605) (Fig. 2), suggesting the less contribution of this chemoattractant to IA development in human or the presence of significant diversity in expression level among cases.

### Expression of CCL3 protein in human IA lesion

**Figure 3:**
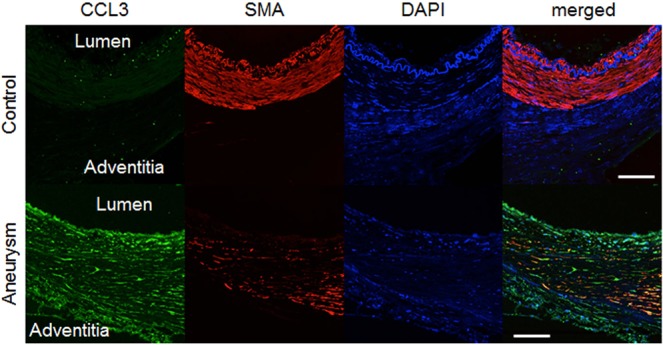
Induced expression of CCL3 protein in intracranial aneurysm lesions. Specimens from control arterial walls (Control) and intracranial aneurysm lesions (Aneurysm) were immunostained. Images of immunostaining for CCL3 (green), α-smooth muscle actin (SMA) as a marker of medial smooth muscle cell (red), nuclear staining by DAPI (blue) and merged ones are shown. Bar, 20 μm. Representative images from 2 (Control) and 4 (Aneurysm) independent specimens are shown in this figure and images from remaining samples are shown in Supplementary Fig. S2.

**Figure 4:**
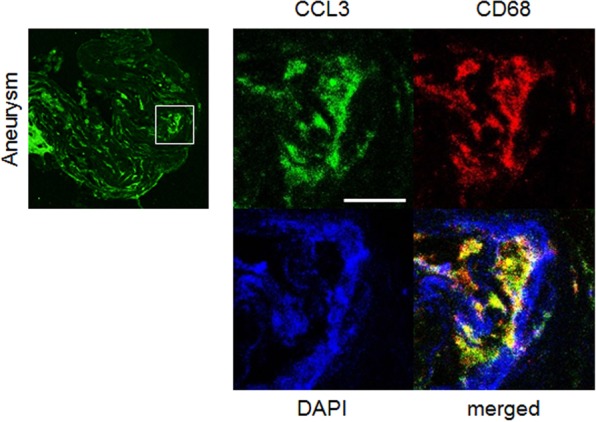
Induction of CCL3 in macrophages of intracranial aneurysm lesions. Images of immunostaining for CCL3 (green), CD68 as a marker for macrophages (red), nuclear staining by DAPI (blue) and merged ones are shown. Square in the left panel indicates the region magnified in right panels. Bar, 10 μm. Representative images from 4 independent experiments are shown.

To clarify that over-expression of CCL3 mRNA content in IA lesions compared with control arterial walls indeed reflects protein expression, we immunostained specimens from 2 control arterial walls and 4 unruptured IAs. While signals for CCL3 were seldom detected in 2 control arterial walls, those signals were abundantly detected as scattered ones in whole arterial walls, including medial smooth muscle cells whose arrangement was disturbed and number was remarkably reduced, of all IA lesions examined (Fig. 3, Supplementary Fig. S2), supporting the result from RNA-sequencing and RT-PCR analyses. Intriguingly, the expression of CCL3 protein in IA lesions was observed also in CD68-positive macrophages (Fig. 4), suggesting the formation of self-amplification loop among this type of cells as similarly demonstrated in a mouse model in which CCL2, not CCL3, mediates formation of amplification loop^19^.

## Discussion

The present experiment revealed the over-expression of genes encoding some chemoattractant factors for macrophages in IA lesions. This finding again highlights the importance of macrophages in the pathogenesis of IAs as indicated by previous studies^14–17^. The contribution of these chemoattractants to maintenance of the persistent macrophage infiltration in lesions and to the pathogenesis of the disease is thus also suggested. Furthermore, the potential of these chemoattractans as therapeutic targets to treat IAs has been indicated. Intriguingly, however, MCP-1 (*CCL2*) is not picked up as an over-expressed gene in human IA lesion. Because we and other researchers have demonstrated the crucial contribution of MCP-1 to the initiation and progression of IAs through recruiting macrophages to form inflammatory environment using animal models^14,15^ and also some have revealed the presence of this type of cells in human IA lesions as a most abundant inflammatory cell *in situ*^16,17^, some hypotheses are now raised. First, because both MCP-1 and CCL3 can recruit macrophages in sites of inflammation under inflammatory settings, usage of these chemoattractant proteins among species may be different. Second, in mice model of IAs, because only IAs at the early stage of the disease can be induced, we can only assess the incidence of lesions. Therefore, MCP-1 and CCL3 may play a role at the different stage of IA formation and progression; i.e. MCP-1 regulates macrophage infiltration to induce IA formation at the early stage and CCL3 functions to progress the disease. Third, expression of MCP-1 and CCL3 depends on cytokine milieu in microenvironment of IA lesions because many factors determine expression of these chemoattractants, i.e. transcriptional regulation by NF-κB, c-Ets or so and post-transcriptional stabilization of mRNA^20^. Thereby, abundance of *CCL2* may be greatly different among lesions which makes difficult to detect statistical significant difference in expression. Here, intriguingly, we demonstrated in our recent manuscript that NF-κB inhibition specifically in endothelial cells in mice led to almost complete suppression of MCP-1 expression in these cells but infiltration of macrophages in the lesions was still sustained^19^ presumably through CCL3 suggesting the redundant role of chemoattractants to recruit macrophages in IA lesions.

Epidemiological study has clarified that only one thirds of IAs in humans ruptures in a lifetime^21^. Further, only a few portion of unruptured IAs annually enlarges and only around 1% of them ruptures^2,22^. These epidemiological findings about natural course of human IAs may imply the nature of unruptured IAs in humans as mostly stable lesions. In line with such an assumption, our RNA sequencing analysis of human unruptured IAs mainly detected changes in gene expression profiles corresponding to loss of medial smooth muscle cells, which is the well-established histological character in IA walls, and only small number of genes was detect as over-expressed ones. Previously reported results from comprehensive gene expression analysis of human IAs have indicated active involvement of inflammatory responses in the pathogenesis of IAs but these studies enrolled mainly ruptured IAs or unruptured ones with a larger size in diameter^8–13^ than a typical one. Because rupture itself supposedly evokes intense inflammation presumably due to marked increase of intracranial pressure and excessive activation of sympathetic nerve system, careful interpretation should be done. Indeed, the published report enrolling only unruptured IAs demonstrated the completely different result^23^. Most typically, annotation of the differentially regulated genes in unruptured IAs includes ‘antigen processing and presentation pathway’, which is picked up as an over-represented pathway in ruptured IAs, as an under-represented one^23^. The present study may suggest that inflammatory responses in IA walls are transiently enhanced, once after inflammation is triggered by hemodynamic force, followed by the relief of inflammatory responses making IA lesions relatively stable. Macrophages CCL3-dependently infiltrated in lesions thereby may exert anti-inflammatory property and contribute such a relief of inflammation. Based on these assumptions, we hypothesize that rupture of IAs requires the resurgence of inflammation by some unrevealed mechanisms as ‘2^nd^ hits’. Potential mechanisms regulating the resurgence of inflammation may be factors previously reported; i.e. thrombus formation^24–26^, lipid accumulation^27^ presumably by excessive endothelial cell damage or occurrence of aberrant flow condition by morphological changes during the progression of IAs^25,28^. Given the clinical studies in which drugs with anti-inflammatory effect, statins or non-steroidal anti-inflammatory drugs (NSAIDs)^29–31^, significantly reduces the risk of rupture of IAs, regardless the trigger of 2^nd^ hits, inflammation may be crucial also for rupture.

The major limitation about the present study is the small sample size partially because the quality and the amount of total RNA purified from surgically resected specimens were often low. Another limitation is the usage of extra-cranial arteries (middle meningeal artery or superficial temporal artery) as a control. Although the usage of intra-cranial arterial walls where IA is formed of course provides best reference data, we used extra-cranial arteries because the resection of intra-cranial arteries results in ischemic stroke (death of neurons). Also importantly, we could prepare control arteries from the same patients with IA lesions if we used extra-cranial arteries. However, there may be a considerable difference in gene expression profile between intra-cranial arteries, where IAs are formed, and extra-cranial arteries. If present, results obtained in the present study are needed to be carefully interpreted. Also, the subtraction of the comprehensive gene expression to minimize effect of the loss of medial smooth muscle cells using a primary culture of human carotid artery may also interfere with an interpretation of data especially because cultured smooth muscle cells seemto have a synthetic phenotype which enhances expression of some pro-inflammatory genes and thereby may attenuate a contribution of inflammation in lesions. Indeed, some GO-terms were lost after the subtraction (Supplementary Tables S2 and S8). Nonetheless, it may be worthy to note that the importance of macrophages in the pathogenesis is highlighted.

## Methods

### Study Approval

The use of human samples in the present research was approved by the Ethics Committe at Kyoto University Graduate School of Medicine (Application Number; #E2540, #G529, #R0456 and #R0601), RIKEN Yokohama Branch (Application Number; H17-34(14)), National Cerebral and Cardiovascular Center (Application Number; #M29-050) and each hospital where samples were prepared (Japanese Red Cross Asahikawa Hospital, Tokyo Women’s Medical University Yachiyo Medical Center, Tokyo Rosai Hospital, Shinkawahashi Hospital, NTT Medical Center Tokyo). All research was performed in accordance with relevant guidelines and regulations. Also the written informed consent was obtained from each case.

### RNA sequencing analysis of human specimen

Human IA samples and control arteries (middle meningeal artery or superficial temporal artery) were obtained during neck clipping of unruptured IAs. Only saccular aneurysms were included in the present study and other than saccular ones such as fusiform aneurysm, dissecting aneurysm and giant thrombosed aneurysm were excluded. RNA was, then, purified from these samples by using a RNeasy Fibrous Tissue Mini Kit (QIAGEN, Hilden, Germany) according to the manufactures’ instructions. After quality check of purified RNA by the RNA analyzer and a Quant-iT RiboGreen RNA Reagent (Molecular Probes, Eugene, OR), the library was prepared using a TruSeq RNA Sample Preparation v2 Kit (Ilumina, San Diego, CA) according to the manufacture’s protocol with some modifications. In brief, after purification of polyA^+^ RNA from total RNA samples, the RNA was transcribed into cDNA using a SuperScriptIII instead of SuperScriptII (Thermo Fisher Scientific Inc., Waltham, MA). The 1^st^ stranded cDNA was subjected into 2^nd^ stranded cDNA synthesis, followed by end repair reaction and A-tailing reaction. After purification with an AMpure XP (Beckman Coulter, Indianapolis, IN), sequencing adapter provided by a Sample Prep kit was ligated to the double stranded DNA. After purification with an AMpure XP (Beckman Coulter), the amount of the adapter-ligated DNA was then quantified by quantitative PCR analysis using KAPA Library Preparation Kits with a Real-Time Amp (Kapa Biosystems Inc., Woburn, MA) to estimate the optimal PCR cycle number for library amplification. The amplification of library was performed using KAPA Library Amplification Kits (Kapa Biosystems Inc.) with optimized PCR cycles. The gene expression profile was then obtained by RNA sequencing (100 bp paired-end read sequencing, HiSeq2500, Illumina). The criteria for RNA quality to perform RNA sequencing analysis are that a sample amount is over 100 ng and the ratio of A260/280 and A260/A230 is both over 1.8 (http://www2.clst.riken.jp/genas/services/rnaseq.html). The raw data was processed by using the MOIRAI workflow system (PMID: 24884663)^32^, where bcl2fastq (version 1.8.4) was used for basecalling and demultiplexing, FASTQC (version 0.11.3) for sequence quality check, fastx_clipper (version 0.0.13.2) for adaptor trimming and tophat (version 2.0.12) for alignment with the human reference genome (hg19). The mapped reads were counted per gene by HT-seq (version 0.6.1 p1, PMID: 25260700). Normalization of gene expression profiles and differential analysis were conducted by using edgeR (version 3.8.6, PMID: 19910308) with false discovery rate 0.01 and two fold change as a threshold for statistical significance, after genes expressed in primary culture of smooth muscle cells from human carotid artery were excluded.

Data from RNA sequencing analysis is deposited to the BioProject database at NCBI (http://www.ncbi.nlm.nih.gov/bioproject) as ID number #PRJNA553307.

### Primary Culture of Vascular Smooth Muscle Cells from human carotid artery

The primary culture of vascular smooth muscle cells from human carotid artery was purchased from Cell Applications (#3514-05a, San Diego, CA, USA) and maintained in the special medium obtained from this company. This primary culture is maintained in a proliferative state and thus may have a synthetic phenotype. The feature of this primary culture as a vascular smooth muscle cell was examined by expression of α-smooth muscle actin (SMA) in immunohistochemistry (Supplementary Fig. S1A). Further, mRNA expression of markers for a smooth muscle cell, SMA (*ACTA2*), Caldesmon (*CALD1*), Calponin1 (*CNN1*) and SM1 (*MYH11*), was examined using the expression of β-actin (*ACTB*) as a positive control by PCR analysis (Supplementary Fig. S1B). Amplified product in each PCR reaction was separated by gel electrophoresis.

Primer sequence used were as follows; forward 5′- CATACTCCTGCTTGCTGATCC-3′and reverse 5′-GATGCAGAAGGAGATCACTGC-3′ for *ACTB*, forward 5′-TGTAGGTGGTTTCATGGATGC-3′ and reverse 5′-GAGTTACGAGTTGCCTGATGG-3′ for *ACTA2*, forward 5′-CCTCTTCATCATCGTCATTCC-3′ and reverse 5′-TCCAGACATCATCTGGTCTCC-3′ for *CALD1*, forward 5′-TACTTCACTCCCACGTTCACC-3′ and reverse 5′-AACGACCTGTTTGAGAACACC-3′ for *CNN1*, and forward 5′-CCTCTTCTGCAAGATTTGTCG-3′ and reverse 5′-AGGCCAAGATCAAGAAACTGG-3′ for *MYH11*.

RNA was purified from these cells at P3 and subjected to RNA sequencing analysis.

### RNA extraction and quantitative real time PCR (RT-PCR) analysis of human specimen

Total RNA from another 18 human specimens than RNA sequencing analyses was purified as described above and reverse-transcribed into cDNA using by a High Capacity cDNA Reverse Transcription Kit (Thermo Fisher Scientific Inc.) according to manufacturer’s instructions. Quantitative RT-PCR analysis was, then, done with a SYBR Premix Ex Taq II (Takara, Shiga, Japan) and a Real Time System CFX96 (Bio-rad, Hercules, CA). For quantification, the second derivate maximum method was used for crossing point determination. The value of each gene expression was normalized to β-actin expression and then the expression in IA lesions was expressed as a relative mRNA expression over expression in a control arterial wall.

Primer sets used in RT-PCR reaction were listed following; forward 5′-AGCTTCTTTGGGACACTTGC-3′, reverse 5′-ATAGCAGCCACCTTCATTCC-3′ for *CCL2*, forward 5′-TGATGCAGAGAACTGGTTGC-3′, reverse 5′-CAGTGGTCAGTCCTTTCTTGG-3′ for *CCL3*, forward 5′-AACAGTGACAGTGGACCATCC-3′, reverse 5′-TCCATACTCAGGACTCCTCTCC-3′ for *CCL4*, forward 5′-GGTAGCCACTGAAAGAATTTGG-3′, reverse 5′-ACCTTTCCCATCTTCCAAGG-3′ for *CXCL10*, forward 5′-AGTGTCTCTTCAGGGCTTTGC-3′, reverse 5′-GGACAGCAGCATTGTTTATGG-3′ for *CLEC5A*.

### Human specimen and immunohistochemistry

Human IA samples and control arterial walls (superficial temporal artery or middle meningeal artery) were dissected during microsurgical clipping of unruptured IAs with the written informed consent. Dissected specimen was fixed in formalin solution and embedded in paraffin. 4-μm thick slices were then prepared for immunohistochemical analysis. After deparaffinization and blocking with 3% donkey serum (Jackson ImmunoResearch, West Grove, PA), slices were incubated with primary antibodies followed by incubation with secondary antibodies conjugated with fluorescence dye (Jackson ImmunoResearch). Finally, fluorescent images were acquired on a confocal fluorescence microscope system (Lsm-710, Carl Zeiss Microscopy GmBH, Gottingen, Germany).

Primary antibodies used were as follows. goat polyclonal anti-CCL3/MIP-1 alpha antibody (R&D Systems, Minneapolis, MN), mouse monoclonal anti-smooth muscle α-actin (SMA) antibody (Thermo Fisher Scientific Inc.), mouse monoclonal anti-CD68 antibody (Abcam, Cambridge, UK).

### Statistical analysis

Data are shown as the mean ± SEM, and 2 groups were statistically compared using the non-parametric Wilcoxon’s test. Statistical comparisons among more than 2 groups were conducted using the non-parametric Kruskal–Wallis test followed by the Steel test. A p value smaller than 0.05 was defined as statistically significant.

## Supplementary information

Supplementary Table S1

Supplementary Table S3

Supplementary Table S4

Online Supplement
