# Supplementary material for: RNA sequencing analysis revealed the induction of CCL3 expression in human intracranial aneurysms
Source: Sci Rep. 2019 Jul 17;9:10387. doi: 10.1038/s41598-019-46886-2 (PMC6637171; doi:10.1038/s41598-019-46886-2)
Supplement: Supplementary file 4 — Online Supplement [file 41598_2019_46886_MOESM4_ESM.docx]

RNA sequencing analysis revealed

the induction of CCL3 expression in human intracranial aneurysms.

Tomohiro Aoki, Hirokazu Koseki, Haruka Miyata, Masayoshi Itoh, Hideya Kawaji, Katsumi Takizawa, Akitsugu Kawashima, Hiroshi Ujiie, Takashi Higa, Kenzo Minamimura, Toshikazu Kimura, Hidetoshi Kasuya, Kazuhiko Nozaki, Akio Morita, Hirotoshi Sano, Shuh Narumiya

**Supplementary Figures**

**
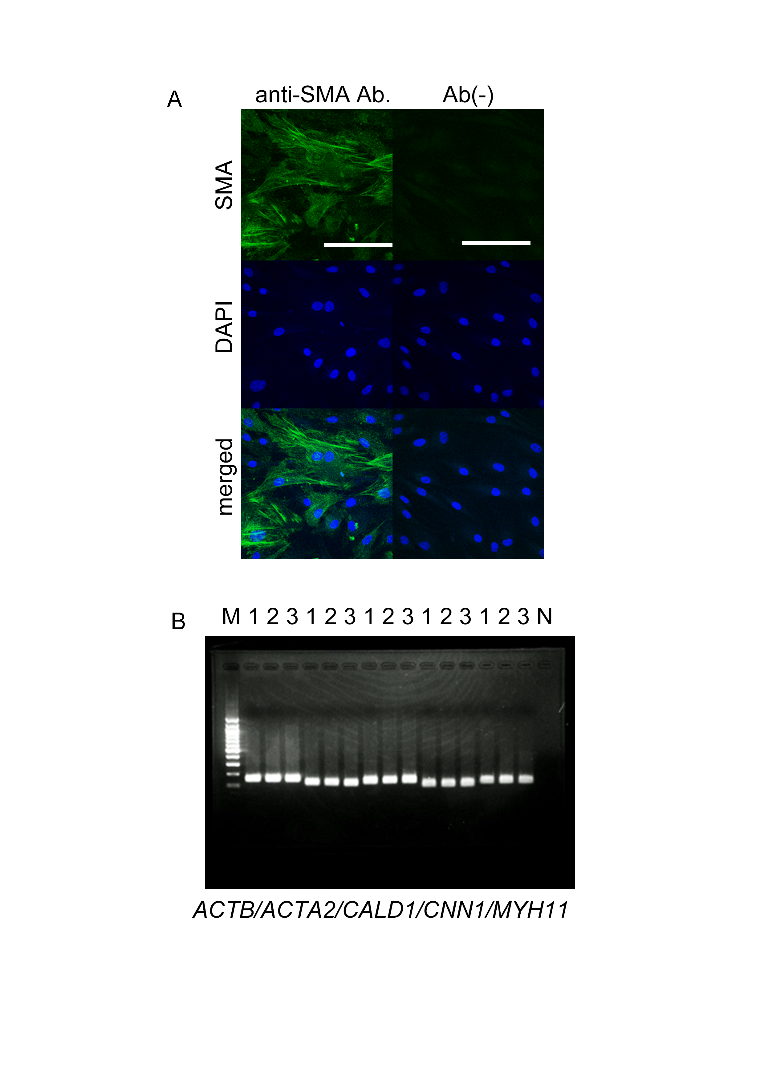
**

**Supplementary Figure S1. Characterization of primary culture of vascular smooth muscle cells from human carotid artery.**

Primary culture of vascular smooth muscle cells were characterized by expression of α-smooth muscle actin (SMA) in immunohistochemistry (A) and by mRNA expression of β-actin (*ACTB*) and characteristic genes for vascular smooth muscle cells, SMA (*ACTA2*), Caldesmon (*CALD1*), Calponin1 (*CNN1*) and SM1 (*MYH11*), in PCR analysis (B). Images of immunostaining for SMA (green), nuclear staining by DAPI (blue) and merged ones are shown in A using immunostaining without a primary antibody as a negative control (Ab (-)). Bar, 30 μm. Representative images from 3 independent experiments are shown. PCR product using cDNA samples (3 samples as indicated 1 to 3) prepared from primary cultured cells and primer sets as indicated was separated by gel electrophoresis and the image of the gel is shown in C. M and N indicate a size marker and a negative control reaction without primers, respectively.

**Supplementary Figure S2. Induction of CCL3 in intracranial aneurysm lesions.**

Specimens from control arterial walls (Ctrl, n=2) and intracranial aneurysm lesions (An, n=4) were immunostained. Images of immunostaining for CCL3 (green), α-smooth muscle actin (SMA) as a medial smooth muscle cell marker (red), nuclear staining by DAPI (blue) and merged one are shown. Immunostained images without primary antibodies are also shown in right panels as a negative control experiment. Images from Hematoxylin-Eosin staining (HE) of an adjacent section of each immunostained one are shown in most upper panels. Bar, 20 μm. Images of Ctrl1 and An3 are used in Figure 3.

**Supplementary Tables**
